# Insights into genetic determinants of volatile fatty acid catabolism in *Cupriavidus necator* H16

**DOI:** 10.1128/aem.00515-25

**Published:** 2025-06-12

**Authors:** Eric C. Holmes, Stephanie L. Breunig, Christopher W. Johnson, Gregg T. Beckham, Alissa C. Bleem

**Affiliations:** 1 Renewable Resources and Enabling Sciences Center, National Renewable Energy Laboratory 53405, Golden, Colorado, USA; Kyoto University, Kyoto, Japan

**Keywords:** waste valorization, *Cupriavidus necator*, metabolic engineering, fatty acid metabolism, gene regulation, acyl-CoA synthetase

## Abstract

The soil bacterium *Cupriavidus necator* H16 is a promising host for upgrading waste-derived volatile fatty acids (VFAs) into renewable biochemicals. While bacterial VFA metabolic pathways are well understood, the *C. necator* genome encodes multiple enzymes for each catabolic step, and the degree of substrate specificity among these homologs is currently unknown. To gain insight into the catabolism of VFA substrates in *C. necator*, we performed transcriptomics on cells grown with acetate, propionate, butyrate, valerate, or hexanoate as the sole source of carbon and energy. These data revealed that *C. necator* upregulates multiple sets of genes putatively involved in substrate activation and *β*-oxidation in response to VFAs. To better understand this redundancy, we performed biochemical and genetic deletion studies of acyl-CoA synthetase enzymes upregulated during growth on VFA substrates. These results demonstrated the functional redundancy of the *C. necator* VFA catabolism and led to the identification of a gene cluster, *H16_B1332-H16_B1337*, that contains several genes that are important for the efficient catabolism of hexanoate. Constitutive expression of a second copy of these hexanoate catabolism genes did not improve growth of *C. necator* on hexanoate, suggesting that other factors (e.g., redox, transport, or toxicity) may be limiting for growth. Collectively, this work provides new insight into how *C. necator* uses metabolic regulation to effectively utilize VFA substrates and uncovers the important role of the gene cluster *H16_B1332-H16_B1337* in the catabolism of hexanoate.

**IMPORTANCE:**

The development of efficient bioprocesses that utilize waste-derived carbon will be important for ensuring the circularity of carbon flows and the sustainability of new biotechnologies. Unfortunately, carbon substrates that can be reliably sourced from waste are often toxic or inefficient growth substrates for industrially relevant bacteria. A more complete understanding of the regulatory and biochemical mechanisms that bacteria use to respond to and catabolize waste-derived carbon resources will enable metabolic engineering strategies to improve bioconversion of these same resources. In this study, we provide new insight into these mechanisms for an emerging and promising host-feedstock pairing: *Cupriavidus necator* H16 and volatile fatty acids (VFAs). We anticipate that these insights can be leveraged in future work to engineer *C. necator* to more efficiently convert VFAs into sustainable protein and bioproducts.

## INTRODUCTION

Biological upgrading of waste-derived carbon substrates will be a key component of the growing bioeconomy. While substantial progress has been made to engineer microbial strains for target molecule production from “first-generation” feedstocks (e.g., glucose), discovery and optimization of pathways for alternative feedstock utilization are comparatively understudied (1). Elucidation of microbial strategies for incorporating additional substrates will enable metabolic engineering efforts to more effectively utilize abundant waste resources. The soil bacterium *Cupriavidus necator* H16 (*C. necator*, formerly *Ralstonia eutropha*, *Alcaligenes eutrophus*, *Wautersia eutropha*, and *Hydrogenomonas eutropha*) has a diverse metabolism and, due to its prior use at industrial scales and relative ease of genetic engineering, is a promising host for optimization of waste-utilization pathways (2–4). Several efforts have utilized *C. necator* to upgrade volatile fatty acid (VFA) substrates (e.g., acetate, propionate, butyrate, valerate, and hexanoate) toward biomass and polyhydroxybutyrate (PHB) production (5–9). VFAs are a particularly promising feedstock because they can be sourced from multiple sustainable processes and abundant waste resources, including electrochemical reduction of waste gases (e.g., CO and CO_2_) (10) and arrested anaerobic digestion of food and agricultural wastes (11, 12). While *C. necator* can utilize VFAs as its sole source of carbon and energy (13), they are not its preferred growth substrate and are noted for their microbial toxicity (9, 14). We previously used adaptive laboratory evolution and metabolic engineering to improve the growth of *C. necator* on VFAs (9), but targeted engineering approaches for improved growth require additional knowledge of the regulatory, biochemical, and metabolic mechanisms that contribute to VFA catabolism.

**Fig 1:**
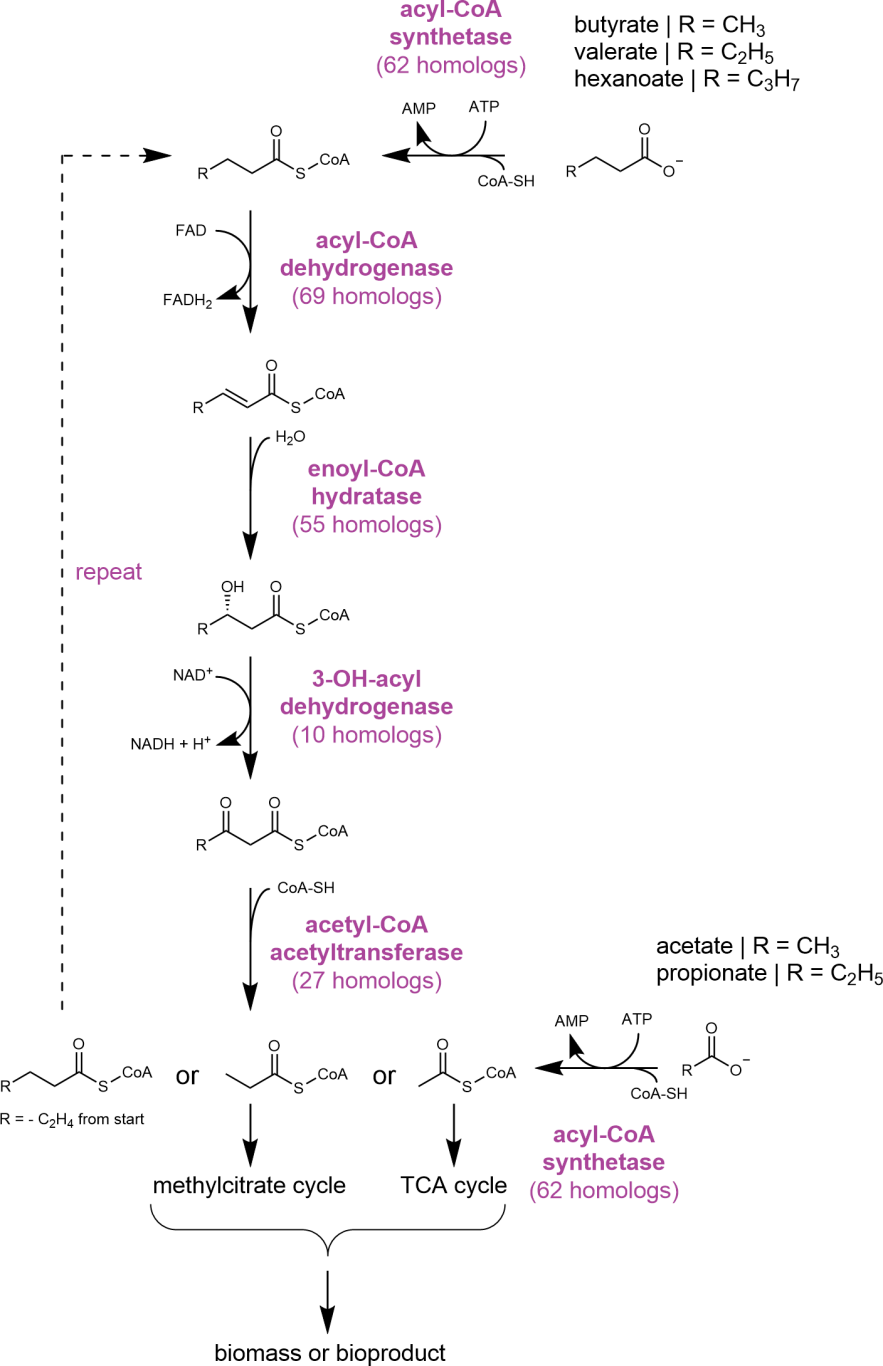
Putative pathway for the catabolism of VFA substrates acetate (**C2**), propionate (**C3**), butyrate (**C4**), valerate (**C5**), and hexanoate (**C6**). Acetate and propionate are activated by conversion to their respective CoA thioesters by acyl-CoA synthetase enzymes prior to entry into central metabolism via the TCA or methylcitrate cycles. Each butyrate, valerate, and hexanoate molecule enters the *β*-oxidation cycle and is converted to two acetyl-CoA molecules, one acetyl-CoA molecule plus one propionyl-CoA molecule, and three acetyl-CoA molecules, respectively. The number of predicted homologs in *C. necator* for each enzymatic reaction is indicated below each protein name. Homolog predictions are based on Kyoto Encyclopedia of Genes and Genomes database annotations (23) and previous studies (16, 17).

*C. necator* is a metabolic generalist notable for its diverse metabolic capabilities (2). While this likely enables *C. necator* to quickly adapt in soil environments, it can be detrimental to its use in industrial settings because the large genome required to enable this flexibility can lead to functional redundancy and non-optimized resource allocation (15). Catabolism of VFAs appears to be an area with considerable functional redundancy, as *C. necator* contains over 60 genes encoding acyl-CoA synthetase (ACS) enzymes (required for catabolism of C2-C6 VFAs) and upwards of 70 putative homologs of genes coding for enzymes in the *β*-oxidation pathway (responsible for catabolism of C4-C6 VFAs) (Fig. 1). Previous studies suggest that multiple operons are involved in the catabolism of longer-chain fatty acids (16–18), yet it is currently unknown whether these genes are important for the catabolism of VFAs. Studies in other promising waste-utilization hosts (e.g., purple non-sulfur bacteria and *Pseudomonas putida*) have provided considerable insight into the metabolic and redox-related factors that are important for VFA utilization (19–21). For example, homologous *β*-oxidation genes are known to display some substrate specificity in *P. putida* (21), so additional knowledge into how *C. necator* regulates expression of these genes when grown on VFAs could provide mechanistic insight into the role of specific genes in the catabolism of specific substrates. This knowledge could enable genome-reduction strategies (22) or metabolic engineering to improve VFA catabolism. Because VFA catabolism requires enzymes from broad classes (e.g., acyl-CoA synthetases and dehydrogenases), an improved understanding of the specificity of these enzymes may also enable efforts to expand the breadth of substrate utilization in diverse microbial hosts.

In this work, we first performed RNA sequencing to determine gene expression profiles of *C. necator* grown on the VFA substrates acetate, propionate, butyrate, valerate, and hexanoate. These data revealed differential gene expression patterns of putative VFA catabolism genes, with some dependency on the VFA chain length. Key among these differences were expression profiles of genes encoding ACSs, which catalyze the formation of acyl-CoA thioesters and are hypothesized to initiate the catabolism of all VFA substrates tested (Fig. 1). Due to their importance as an entry-point reaction for diverse metabolic pathways, we chose to investigate the specificity of ACS enzymes in more detail using *in vitro* enzyme assays and functional gene deletions. These experiments revealed genetic and functional redundancies in the ACS activity for all tested VFA substrates aside from hexanoate, where the gene *H16_B1335* appeared to be the key for hexanoate catabolism. This result, coupled with previous work demonstrating that hexanoate can be selectively produced and isolated during waste conversion processes, led us to characterize hexanoate catabolism in more detail (24, 25). Functional characterization of genes co-localized with *H16_B1335* led to the discovery of a gene cluster, including biosynthetic and regulatory genes required for efficient growth of *C. necator* on hexanoate. Expression of a second copy of these genes at a different genomic site did not improve growth on hexanoate, suggesting that other metabolic or physiological factors limit growth under tested conditions. Additional copies of these genes, however, did modestly improve the growth rate of *C. necator* on valerate, highlighting the functional redundancy of genes involved in VFA catabolism. Together, this work provides important insight into the regulatory and metabolic factors that enable VFA catabolism in the industrially relevant microbe *C. necator*.

## RESULTS

### RNA sequencing of *C. necator* provides insight into VFA catabolism

To better understand the regulatory, biochemical, and metabolic factors that are important for catabolism of VFAs in *C. necator*, we performed transcriptomics on cells grown using C2–C6 linear-chain VFAs (acetate, propionate, butyrate, valerate, and hexanoate) as the sole carbon source (Tables S1 and S2). We also included two control substrates for growth: fructose and malate. Because malate is a tricarboxylic acid (TCA) cycle intermediate, we hypothesized that its catabolism would cause minimal perturbations to peripheral metabolic pathways, enabling us to effectively compare the putative aspects of VFA catabolism. For these experiments, we used a previously constructed strain of *C. necator* that contains several genetic modifications to enable genetic engineering and improved growth on heterotrophic substrates (CHC123, Table 1; Table S3) (22). CHC123 and all other strains in this study have the restriction enzyme encoded by *H16_A0006* deleted to improve transformation efficiency (26) and the operon *phaCAB* deleted to prevent production of the storage polyester polyhydroxybutyrate (PHB). The megaplasmid pHG1 was also deleted in these strains since it accounts for 6% of the genome but is dispensable for, and in some cases detrimental to, growth on most heterotrophic substrates (22).

**TABLE 1:** *C. necator* strains in this study^*a*^

Strain	Genotype	Source
CHC123	*Cupriavidus necator* ATCC 17699 Δ*H16_A0006* Δ*pHG1* Δ*phaCAB*	(22)
ERH090	*Cupriavidus necator* ATCC 17699 Δ*H16_A0006* Δ*pHG1* Δ*phaCAB* Δ*phaR* Δ*H16_A1373*	(9)
ERH095	CHC123 Δ*H16_A1358*	This work
ERH096	CHC123 Δ*H16_B1335-H16_B1337*	This work
ERH105	CHC123 Δ*H16_B1102*	This work
ERH107	CHC123 Δ*H16_B1148*	This work
ERH108	ERH090 Δ*H16_B1335-H16_B1337*	This work
ERH116	CHC123 Δ*H16_A0285*	This work
ERH118	CHC123 Δ*H16_B1335*	This work
ERH120	ERH090 Δ*H16_A0285*	This work
ERH122	ERH090 Δ*H16_B1335*	This work
ERH124	ERH090 Δ*H16_A1358*	This work
ERH126	ERH090 Δ*H16_B1102*	This work
ERH128	ERH090 Δ*H16_B1148*	This work
ERH130	CHC123 Δ*H16_B1332*	This work
ERH168	ERH090 Δ*H16_B1332*	This work
ERH172	ERH090 Δ*pH16_B1335::pTac*	This work
ERH177	CHC123 Δ*H16_B1336-H16_B1337*	This work
ERH178	ERH090 Δ*H16_B1336-H16_B1337*	This work
ERH180	CHC123 Δ*H16_B1334*	This work
ERH183	ERH090 Δ*H16_B1334*	This work
ERH184	CHC123 Δ*H16_B1332-H16_B1337*	This work
ERH196	CHC123 Δ*H16_B1264*	This work
ERH198	ERH090 Δ*H16_B1264*	This work
ERH201	ERH090 Δ*H16_A0006::pTac_H16_B1335* Δ*H16_B1335*	This work
ERH203	CHC123 Δ*H16_A3514*	This work
ERH205	ERH090 Δ*H16_A3514*	This work
ERH210	ERH090 Δ*H16_B1332-H16_B1337*	This work
ERH232	CHC123 Δ*H16_B1339*	This work
ERH234	CHC123 Δ*H16_B1342*	This work
ERH237	ERH090 Δ*H16_B1339*	This work
ERH238	ERH090 Δ*H16_B1342*	This work
ERH280	ERH090 Δ*H16_A0006::pTac_H16_B1332_deOpt - H16_B1335_deOpt - H16_B1334_deOpt*	This work

^*a*^ Corresponding gene annotations can be found in Table S3.

**Fig 2:**
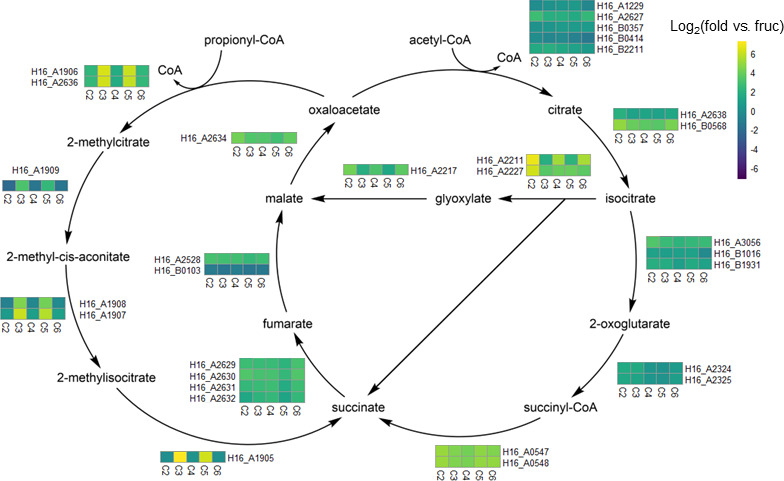
Expression heatmaps for genes of the TCA and methylcitrate cycles projected onto a metabolic pathway map. The position of heatmaps indicates the putative function of the indicated genes. Each heatmap column represents the log-transformed gene expression for the indicated substrate (C2 = acetate, C3 = propionate, C4 = butyrate, C5 = valerate, C6 = hexanoate) compared to fructose (fruc) as a control. All log-transformed relative expression values are plotted on a linear gradient from −7.2 to 7.2 as indicated in the legend on the right of the figure. Cofactors and coenzymes in each pathway are not shown. All data are the average of biological triplicates.

Acetyl-CoA, a central intermediate that is generated from acetate, butyrate, valerate, and hexanoate catabolism, directly enters central metabolism via the TCA cycle (Fig. 1). Hence, we found that genes encoding enzymes of the TCA cycle were generally upregulated during growth on VFAs compared to fructose (Fig. 2; Table S4). Specifically, aconitase (*H16_B0568*), succinyl-CoA synthetase (*H16_A0547/H16_A0548*), and malate dehydrogenase (*H16_A2634*) were the highest upregulated TCA cycle genes (Fig. 2; Table S4). Genes of the glyoxylate shunt (isocitrate lyase [*H16_A2211* and *H16_A2227*] and malate synthase [*H16_A2217*]) were also highly upregulated across all substrates and expressed most highly for even-chain VFAs (Fig. 2; Table S4). Propionyl-CoA, a central intermediate that is generated during propionate and valerate catabolism, enters central metabolism via the methylcitrate cycle (Fig. 1). Genes encoding enzymes of the methylcitrate cycle were specifically upregulated on these odd-chain VFAs (propionate and valerate) (Fig. 2; Table S4). These results suggest that *C. necator* effectively regulates central metabolic pathways required for catabolism of even- versus odd-chain VFAs.

The genome of *C. necator* contains many genes that are putatively involved in fatty acid metabolism, indicating some functional redundancy for the steps of fatty acid activation and *β*-oxidation (Fig. 1) (16–18). However, deletion of several clusters containing genes of the classes required for *β*-oxidation has led to strains with growth defects on long- and medium-chain fatty acids, suggesting an important role for these genes in the catabolism of fatty acid substrates (16–18). We found that genes of two of these clusters were only slightly upregulated on C2–C6 VFA substrates (*H16_A0459-H16_A0464* and *H16_A1526-H16_A1531*), suggesting they instead have a more prominent role in the catabolism of longer-chain fatty acids (Fig. S1A). Conversely, *H16_B1187-H16_B1192* was highly upregulated on propionate and butyrate, suggesting its role in their catabolism (Fig. S1A). However, even though these genes were previously studied in fatty acid catabolism (16–18), they were generally not the top overexpressed genes related to *β*-oxidation in our experiments with VFA substrates. Instead, other heretofore uncharacterized genes putatively related to *β*-oxidation and fatty acid activation were more upregulated, and we hypothesized that they may be required for the catabolism of VFAs (Fig. S2 and S3; Table S4). Additionally, we observed that different sets of putative fatty acid catabolism genes were upregulated on different VFA substrates (Fig. S3; Table S4). To better understand how these upregulated genes enable efficient catabolism of VFAs, we focused our initial characterization efforts on the first step required for metabolism of C2–C6 VFA substrates, namely, activation by conversion to their respective -CoA thioester.

### *In vitro* characterization of ACS enzymes provides insight into substrate specificity

**Fig 3:**
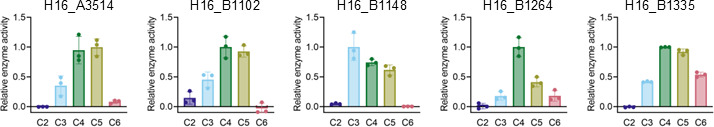
Relative activity of the ACS enzymes purified from *E. coli* and tested *in vitro* on the substrates acetate (C2, dark blue bars), propionate (C3, light blue bars), butyrate (C4, green bars), valerate (C5, gold bars), and hexanoate (C6, pink bars). Values are normalized by the average specific activity (µM ATP consumed/µg protein/min) of the most active substrate for each respective enzyme. Bars represent the average, and error bars represent the standard deviation of biological triplicates for each ACS enzyme. Statistical comparisons are provided in Fig. S4D. Un-normalized specific activity values are displayed in the same format in Fig. S4E.

ACS and acyl-CoA transferases (ACT) catalyze the thioesterification of carboxylic acids, and this ubiquitous reaction serves as an essential activation step for the catabolism of diverse carbon substrates, including aromatic carboxylates, long-chain fatty acids, and VFAs (Fig. 1) (27–29). We found that several ACS- and ACT-encoding genes were highly upregulated on VFA substrates compared to fructose and malate (Fig. S1B and C), and based on this observation, we hypothesized that *C. necator* may tailor its biosynthetic response to VFA substrates by expressing ACS and ACT genes with substrate specificity. To identify substrate preference for the most highly upregulated ACS and ACT enzymes (Fig. S1B and C), we heterologously expressed, purified, and measured the relative activity of selected enzymes using *in vitro* enzyme-coupled assays (Fig. S4A). All five tested enzymes were active on VFA substrates, with most showing the highest relative activity on C3 (propionate) through C5 (valerate) substrates (Fig. 3; Fig. S4). The relative activity of H16_B1148 skewed toward shorter chain lengths, while the relative activity of H16_B1335 skewed toward longer chain lengths, mirroring the gene expression pattern that was observed with these substrates (Fig. 3; Fig. S4B and C). Generally, enzymes that were active on specific substrates were also upregulated on those substrates, but the trends in normalized activity did not always match the relative gene expression (e.g., *H16_B1102* was most highly overexpressed on propionate but was most active *in vitro* on butyrate and valerate) (Fig. 3; Fig. S4B and C). Collectively, these results confirm that, for a given VFA substrate, the ACS genes upregulated by *C. necator* during growth encode enzymes that are catalytically active on the same VFA.

### The ACS gene *H16_B1335* is important for growth of *C. necator* on hexanoate

**Fig 4:**
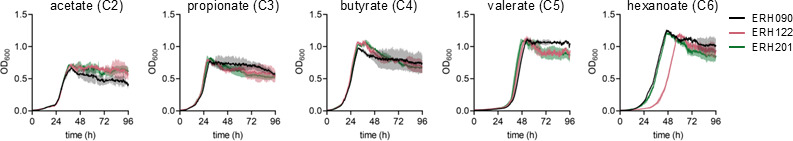
Growth measured as OD_600_ of ERH090 (parent strain, black curves), ERH122 (*H16_B1335* deletion strain, pink curves), and ERH201 (*H16_B1335* complementation strain, green curves) grown on acetate, propionate, butyrate, valerate, and hexanoate as indicated above individual graphs. Lines represent the average, and shading represent the standard deviation of biological triplicates.

To gain additional insight into the role of specific ACS and ACT enzymes in metabolism of VFAs, we individually deleted seven ACS and ACT genes from CHC123 and from a previously constructed strain of *C. necator* that was engineered for improved growth on VFAs (ERH090) (9) (Table 1). ERH090 harbors mutations to genes that significantly impact VFA catabolism and redox regulation, and we hypothesized that studying the function of ACS and ACT genes in both strain backgrounds could reveal different genetic dependencies (9). All 14 strains were tested for growth with single linear VFAs (C2–C6) as the sole source of carbon and energy (Fig. 4; Fig. S5 and S6). Of the 70 deletion/substrate conditions tested, only deletion of *H16_B1335* from the ERH090 background strain (strain ERH122) showed a significant growth defect—a significant lag increase compared to the parent strain—and this defect only occurred when ERH122 was grown on hexanoate, suggesting that H16_B1335 is a key enzyme in hexanoate metabolism (Fig. 4). The deletion strain ERH122 was complemented by expressing H16_B1335 driven by a heterologous constitutive promoter at the *H16_A0006* locus. This strain, ERH201, restored growth on hexanoate at concentrations of 20 and 30 mM, providing additional evidence that the growth defect in ERH122 is driven by a lack of H16_B1335 activity (Fig. 4; Fig. S4F). The failure of the other gene deletions to cause significant growth defects is likely the result of metabolic compensation by redundant gene products, supporting our findings that multiple overexpressed ACS genes are active on propionate, butyrate, and valerate (Fig. 3). It is likely that similar compensatory genes eventually enable growth in ERH122, but these gene products may be less active or require a longer lag time for induction. Given the apparent importance of *H16_B1335* to catabolism of hexanoate, we performed additional characterization of the genes surrounding *H16_B1335*.

### The gene cluster *H16_B1332-H16_B1337* encodes proteins important for hexanoate catabolism

**Fig 5:**
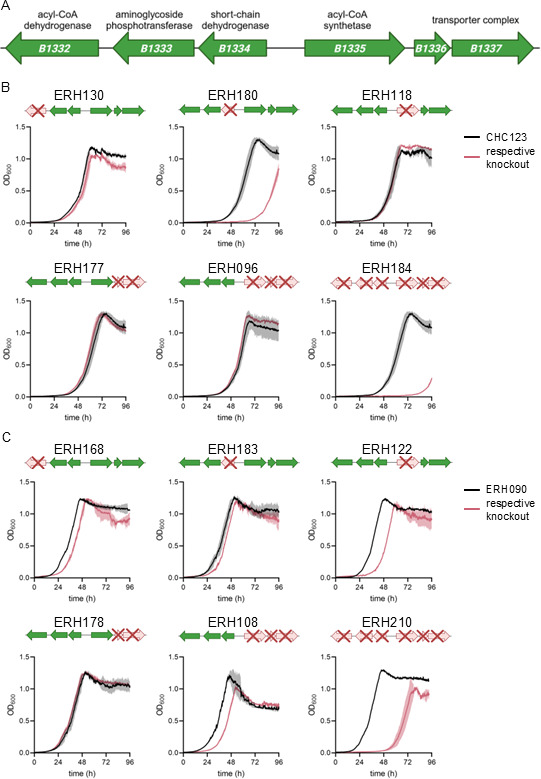
(**A**) Structure of the *H16_B1332-H16_B1337* gene cluster, including putative gene annotations. (**B**) Growth measured as OD_600_ of CHC123 (black curves) and respective gene KOs in the CHC123 strain background (pink curves) grown on hexanoate. (**C**) Growth measured as OD_600_ of ERH090 (black curves) and respective gene KOs in the ERH090 strain background (pink curves) grown on hexanoate. In (**B**) and (**C**), respective gene KOs and strain IDs are indicated above individual graphs. Lines represent the average, and shading represents the standard deviation of biological triplicates.

In the genome of *C. necator*, the ACS gene *H16_B1335* is co-localized with several other genes that are highly overexpressed during growth on hexanoate, including those annotated to encode an acyl-CoA dehydrogenase (*H16_B1332*), a short chain dehydrogenase (*H16_B1334*), and a transporter complex (*H16_B1336-H16_B1337*) (Fig. 5A; Fig. S3E). Individual genes of this cluster were deleted from both CHC123 and ERH090 in several combinations (Table 1), and strains were grown on hexanoate to determine changes in growth. Growth defects were dependent on the specific strain background, suggesting that the regulatory changes induced by mutations to *H16_A1373* and *phaR* in ERH090 may alter expression of compensatory genes and/or change bottlenecks in hexanoate catabolism (Fig. 5). The greatest growth defect caused by a single gene mutation in CHC123 was induced by deletion of the short-chain dehydrogenase H16_B1334 (Fig. 5B), suggesting that CHC123 may be limited by the 3-OH-acyl dehydrogenase step of *β-*oxidation (Fig. 1). Conversely, other paralogous genes and pathways could compensate for this limitation in ERH090, where deletion of H16_B1334 has little effect, and the strain is most severely impaired by deletion of the ACS H16_B1335 (Fig. 5C). Collectively, individual gene deletions of *H16_B1332*, *H16_B1334*, and *H16_B1335* led to considerable growth defects in at least one of the two strain backgrounds, indicating that these genes are involved in hexanoate catabolism (Fig. 5). Deletion of the transporter genes *H16_B1336-H16_B1337* did not impact growth in either strain background, suggesting that these genes are not essential or that transport is not growth-limiting under these conditions (Fig. 5). Deletion of the entire gene cluster led to the most severe growth delay in each strain background, indicating a key and potentially cooperative role for these gene products during growth on hexanoate (Fig. 5). Growth defects were specific to hexanoate, as growth of all of these deletion strains was similar to growth of the parent strain when acetate, propionate, butyrate, or valerate was used as substrates (Fig. S7 and S8).

### An adjacent transcription factor regulates expression of *H16_B1332-H16_B1337*

Transcription factors (TFs) are often co-localized with the metabolic genes that they regulate (30), and we hypothesized that TFs adjacent to *H16_B1332-H16_B1337* may be involved in its regulation and therefore be important for activating hexanoate catabolism. There are two TF genes within 10 kb on either side of *H16_B1332-H16_B1337: H16_B1339* (a LysR-family TF) and *H16_B1342* (a LuxR-family TF) (Fig. S9A). Both adjacent TF genes were highly overexpressed in CHC123 when grown on hexanoate, further suggesting that they may play a role in hexanoate catabolism (Table S1). To determine their respective roles, we individually deleted both TF genes from CHC123 and ERH090 (Table 1) and grew these strains on C2–C6 linear VFAs. Deletion of *H16_B1339* did not impact growth; however, deletion of *H16_B1342* significantly impaired growth on hexanoate in both strain backgrounds (Fig. S9). This suggests that *H16_B1342* is important for activating hexanoate catabolism and may be involved in regulating the expression of *H16_B1332-H16_B1337*.

To validate that *H16_B1342* is involved in regulating the expression of *H16_B1332-H16_B1337*, we quantified relative transcript abundance using qRT-PCR in ERH090 and ERH238 cells incubated in medium containing hexanoate. *H16_B1334* and *H16_B1335* were chosen as representative genes because both deletions led to significant growth delays on hexanoate, and they are in separate co-transcriptional units within the gene cluster (Fig. 5A). As hypothesized, expression of both *H16_B1334* and *H16_B1335* was significantly decreased in ERH238 relative to ERH090, suggesting that *H16_B1342* acts as a positive regulator of their expression (Fig. S9D).

### Constitutive expression of genes in the *H16_B1332-H16_B1337* cluster does not improve growth on hexanoate

**Fig 6:**
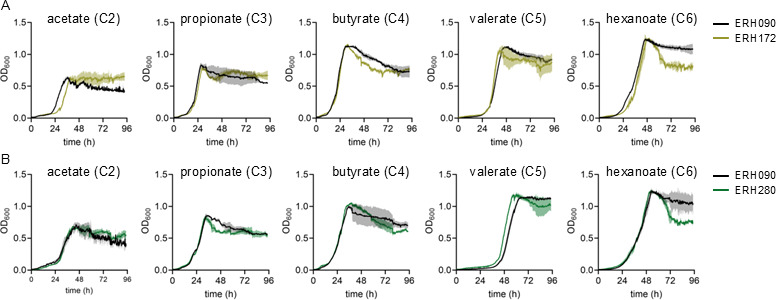
(**A**) Growth measured as OD_600_ of ERH090 (black curves) and ERH172 (gold curves) grown on acetate, propionate, butyrate, valerate, and hexanoate. (**B**) Growth measured as OD_600_ of ERH090 (black curves) and ERH280 (green curves) grown on acetate, propionate, butyrate, valerate, and hexanoate. Lines represent the average, and shading represent the standard deviation of biological triplicates.

The expression of genes in the *H16_B1332-H16_B1337* cluster is highly dependent upon the presence of hexanoate (Fig. S3E), and we hypothesized that constitutive expression of these genes would eliminate the need for this regulatory switch and may enable a more rapid adaptation to growth on hexanoate after preculture on a more preferred substrate (e.g., malate). To test this hypothesis, we first replaced the native promoter of *H16_B1335-H16_B1337* with the high-expression constitutive promoter pTac (31) to generate ERH172. In a second strategy, we integrated a synthetic operon containing *H16_B1332*, *H16_B1334*, and *H16_B1335* driven by pTac at the *H16_A0006* locus to generate ERH280. As a counter to our hypothesis, neither of these constitutive expression strategies decreased lag time or improved growth rate on hexanoate, suggesting that other biochemical factors may be limiting for growth (Fig. 6). We did, however, observe a modest, yet statistically significant, maximum growth rate increase of both ERH172 and ERH280 when grown on valerate (Fig. 6: Fig. S10). This growth rate increase is unsurprising given our *in vitro* assay results demonstrating that *H16_B1335* is also active on valerate (Fig. 3). Collectively, these results demonstrate that the expression of the requisite biosynthetic enzymes may not be the limiting factor for growth on hexanoate under these conditions, and they highlight the need to consider metabolic and physiological factors holistically when optimizing growth on waste-derived substrates.

## DISCUSSION

The ability of *C. necator* to metabolize diverse carbon sources is likely enabled by regulatory systems that respond to potential growth substrates (2). By performing RNA sequencing on a suite of structurally related VFAs, we were able to gain insight into the specificity of these regulatory capabilities in the context of a PHA-deficient strain background. While certain aspects of metabolism were collectively upregulated in response to VFAs (e.g., the TCA cycle and putative VFA catabolic genes) (Fig. 2), we observed several instances of remarkably specific regulatory changes in response to the VFA chain length.

In some cases, these regulatory changes manifested in pathways adjacent to the TCA cycle. We found that genes of the methylcitrate cycle, which converts propionyl-CoA into the TCA cycle intermediate succinate, were specifically upregulated during growth on propionate and valerate (Fig. 2). Propionate and valerate are the only tested substrates that share propionyl-CoA as an intermediate, which suggests that *C. necator* can specifically respond to odd-chain length substrates that require the methylcitrate cycle for their catabolism. Similarly, we found that genes of the carbon-conserving glyoxylate shunt were more highly upregulated on acetate, butyrate, and hexanoate (Fig. 2), which aligns with substrates that exclusively enter the TCA cycle through acetyl-CoA as an intermediate (Fig. 1).

We also found that upregulation of the *H16_B1332-H16_B1337* gene cluster was remarkably specific for cells grown on hexanoate (Fig. S3E). The five most overexpressed genes during growth on hexanoate were in this gene cluster, and these same genes were not significantly upregulated on acetate, propionate, butyrate, or valerate (Fig. S3E). Previous RNA sequencing data gathered on C8 and C9 fatty acid substrates also did not show overexpression of this operon (17), further demonstrating how these genes are specifically expressed in the presence of hexanoate. These expression data and our gene deletion data demonstrating that this operon is important for the growth of *C. necator* on hexanoate (Fig. 5) provide strong evidence that *C. necator* can effectively sense and respond to hexanoate as a growth substrate. Our data suggest that the transcription factor *H16_B1342* is an important component of this regulatory system (Fig. S9), and future work could investigate upstream components of this sensing and signaling network. Gene expression analysis in a *H16_B1342*-deficient background could also shed light on compensatory effects related to the functional redundancy of genes involved in hexanoate catabolism. The potent and specific gene induction regulated by *H16_B1342* could also be leveraged to generate biosensors or regulatory circuits that can respond to hexanoate. This new regulatory and biosynthetic knowledge has considerable translational potential, given that conversion processes have been developed to selectively produce, isolate, and purify hexanoate from low-solid waste inputs (24, 25). In addition to hexanoate, our studies revealed specific gene expression patterns during growth on other VFAs (Fig. S3), and we anticipate that further investigation into these genes may, similarly, lead to new important insights into the specificity of genes required for the catabolism of other VFAs in *C. necator*.

While we did observe some regulatory and biochemical specificities in hexanoate catabolism, our data indicate considerable redundancy in the catabolism of VFAs. Of the 70 deletion/substrate combinations that we tested, only one (ERH122 grown on hexanoate) showed any growth defect (Fig. S5 and S6). Even in cases where deletion of hexanoate catabolism and regulatory genes led to severe lag times (Fig. 5; Fig. S9), exponential growth at a similar rate was eventually achieved, suggesting that regulatory or biochemical changes were eventually able to compensate for the loss of these gene products. This finding reinforces comparative genomics studies on the *Cupriavidus* genus that suggest chromosome 2 (which contains the *H16_B1332-H16_B1337* gene cluster) emerged from repeated lateral gene transfer and duplication events, resulting in considerable redundancy in gene function (32). Our gene deletion results are also supported by our *in vitro* biochemical data that showed each of the five ACS enzymes tested could effectively accept multiple VFA substrates (Fig. 3). Interestingly, the enzymes we tested were most active on C3–C5 substrates, and additional structural characterization may provide insight into how active site architectures may influence substrate preference. Collectively, these results not only highlight the challenge of discovering gene function in species with considerable genetic redundancy but also demonstrate how a combinatorial approach using gene expression, deletion, and *in vitro* activity data can be used to identify ACS enzymes important for VFA catabolism. This strategy may extend to the identification of ACS genes important for the catabolism of other carbon substrates, especially in organisms that exhibit less genetic redundancy than *C. necator*.

While our gene deletion data strongly support the hypothesis that the *H16_B1332-H16_B1337* gene cluster is important for hexanoate catabolism, our strategies to overexpress components of the gene cluster did not lead to improved growth on hexanoate. These results support the notion that other factors can be just as important for catabolism as the robust expression of the requisite biosynthetic machinery. Indeed, we observed this in our previous efforts to improve the growth of *C. necator* on VFAs, where growth improvements were primarily tied to a rebalancing of redox active nucleotides (9). It is possible that strain ERH090 still faces redox- or toxicity-based growth limitations, which would limit the impact that overexpressing the hexanoate catabolic pathway has on growth rate. Given that hexanoate catabolism and PHB biosynthesis are linked via shared metabolites and redox cycling pathways (9), it is important to note that the results of this study may still differ in the context of PHB^+^ *C. necator* strains that may not face the same redox limitations as CHC123, ERH090, and their derivatives. Previous work has shown that PHB biosynthesis and carbon metabolism are linked in diverse bacterial species (9, 33–37), and additional biochemical characterization, like the work we performed here, could help deconvolute and understand these observations. Collectively, our results contribute to our understanding of VFA catabolism in *C. necator* and highlight the importance of considering multiple factors, including biosynthetic machinery, redox balance, and toxicity, when optimizing strains for growth on non-optimal, waste-derived substrates.

## MATERIALS AND METHODS

### Growth media and conditions

*Escherichia coli* strains were grown at 37°C in lysogeny broth (LB) or on LB agar plates with 50 µg/mL kanamycin added for plasmid-harboring strains. *C. necator* strains were grown in LB, modified ATCC 550 R 8 A H medium (1.25 g/L (NH_4_)_2_SO_4_, 0.2 g/L MgSO_4_·7H_2_O, 0.07 g/L CaCl_2_·2H_2_O, 0.01 g/L ferric citrate, 0.02 g/L ethylenediaminetetraacetic acid, 0.6 g/L KH_2_PO_4_, 0.9 g/L K_2_HPO_4_, 1 mL/L trace element solution [1.2 g/L ferric citrate, 20 mg/L MnSO_4_·H_2_O, 10 mg/L H_3_BO_3_, 10 mg/L CuSO_4_·5H_2_O, 20 mg/L (NH_4_)_6_Mo_7_O_24_·4H_2_O, 10 mg/L ZnSO_4_, 0.5 g/L ethylenediaminetetraacetic acid, and 200 mg/L CaCl_2_·2H_2_O], and 750 µL/L vitamin solution [2 g/L nicotinic acid, 2 g/L nicotinamide, 4 g/L thiamine HCl, and 80 mg/L biotin]) (9), or a mixture of 1/2 LB and 1/2 ATCC 550 medium at 30°C. Specific media compositions and growth conditions used for experiments are outlined in the respective method sections.

### Transcriptomics growth and harvest

Transcriptomics growth and harvest were performed as previously described, with minor modifications (9). CHC123 (Table 1) was struck onto LB agar and grown at 30°C for 24 h. The bulk biomass from this plate was used to inoculate two 40 mL cultures in baffled flasks containing ATCC 550 + 20 mM malate medium, which were grown at 30°C with 225 RPM shaking for 16 h. The two 40 mL cultures were combined, centrifuged at 4,000 *× g* for 10 min and washed twice with PBS, and normalized in ATCC 550 medium without carbon to an OD_600_ = 0.5. Two milliliters of this normalized preculture was used to inoculate baffled flasks containing ATCC 550 medium with 20 mM malate, 40 mM fructose, 40 mM acetate, 20 mM propionate, 20 mM butyrate, 20 mM valerate, or 20 mM hexanoate, and cultures were grown at 30°C and 225 RPM. The starting OD_600_ of these cultures was 0.05. Growth was monitored periodically using OD_600_ measurements, and cell material was harvested when cultures first reached mid-log growth (measured OD_600_ between 0.4 and 0.9). For the harvest of material for RNA sequencing, cells were centrifuged at 4,000 × *g* for 3 min; supernatant was removed; and cells were flash-frozen in liquid N_2_ and stored at −80°C. Frozen cell pellets were provided to Azenta Life Sciences, where RNA extraction, library preparation, and RNA sequencing were performed. Illumina HiSeq with 2 × 150 bp reads was used for sequencing at a depth of >25 M reads per sample.

### Transcriptomics data analysis

Transcriptomics data were analyzed as previously described (9). Briefly, data were analyzed using the Department of Energy Systems Biology Knowledgebase (KBase) (38) using a standard transcriptomics analysis pipeline. This included the following programs run in sequence using default parameters: Trimmomatic (39), Bowtie2 (40), StringTie (41), and DESeq2 (42). The *C. necator* H16 genome (AM260480) was used as a reference sequence. Differential expression and TPM outputs from this analysis can be found in Tables S1 and S2. RNA quality parameters (RNA integrity number and DV200) and sequencing statistics can be found in Table S5.

### Plasmid construction for *C. necator* gene deletions and insertions

All gene deletions and insertions were constructed using the conjugative pK18msB plasmid (GenBank accession no. OK423783, Addgene plasmid #177839) (43). pK18msB was first linearized using plasmid-specific primers (Table S6). Inserts synthetized by Twist Biosciences were assembled into linearized pK18msB using NEBuilder HiFi Assembly Master Mix (New England Biolabs) and cloned directly into *E. coli* S17-1 using electroporation. Positive clones were selected using colony PCR with plasmid-specific primers (Table S6) and Phire polymerase (Thermo Scientific), and Oxford Nanopore sequencing was used to verify full plasmid sequences (Plasmidsaurus). All plasmids used in this study are noted in their respective *E. coli* strains in Table S7.

### Generation of *C. necator* strains

Genotypes for all *C. necator* strains constructed in this study are listed in Table 1. *C. necator* strains were constructed as previously described (9, 22). Briefly, *E. coli* S17-1 donor strains harboring the pK18msB vector and *C. necator* parent strains were grown overnight in LB + 50 µg/mL kanamycin and LB medium, respectively, at 30°C with 225 RPM shaking. After approximately 24 h of growth, cultures were concentrated by centrifugation at 4,000 *×g* for 5 min and resuspension in 1/4 volume LB medium. A single *E. coli* donor strain and *C. necator* recipient strain were then combined at a 1:1 volumetric ratio and concentrated by centrifugation at 4,000 *×g* for 5 min and resuspension in 1/4 volume LB medium. Next, 50 µL of this cell mixture was spotted on LB agar, left to dry at room temperature, and incubated at 30°C for 24 h. Then, 1/2 of each spot was restruck onto LB medium with 15 µg/mL gentamycin and 200 µg/mL kanamycin to select for positive *C. necator* conjugants. Individual conjugant colonies were restruck onto LB medium with 15 µg/mL gentamycin and 200 µg/mL kanamycin and grown at 30°C for 24 h to ensure propagation of the plasmid insertion through the genome. Cells were then restruck onto yeast tryptone sucrose agar plates (10 g/L tryptone, 5 g/L yeast extract, 150 g/L sucrose, 18 g/L agar) for counterselection and grown at 30°C. After approximately 3 days, individual colonies were struck onto LB agar and LB agar with 15 µg/mL gentamycin and 200 µg/mL kanamycin. Cell streaks that did not grow on selection medium were screened for gene deletion or insertion using genome-specific primers (Table S6) and Phire polymerase (Thermo Scientific).

For the generation of ERH201, the native gene sequence of *H16_B1335* was inserted into the genomic locus of *H16_A0006* driven by the high-expression constitutive promoter pTac (31) and a high-expression synthetic RBS (44). For the expression of second copies of *H16_B1332*, *H16_B1334*, and *H16_B1335* in ERH280, codon usage was changed to avoid integration of plasmids into the native gene loci. To do so, protein sequences were codon-optimized for expression in *E. coli* using Integrated DNA Technologies’ codon optimization tool (https://www.idtdna.com/CodonOpt) (Table S8). These genes were inserted into the *H16_A0006* locus driven by the same promoter and RBS as in ERH201 using three consecutive conjugation cycles with three separate *E. coli* conjugative donors (Table S7). For the generation of ERH172, 100 bp upstream of *H16_B1335* was removed and replaced with the same high-expression constitutive promoter pTac and synthetic RBS used in ERH201 and ERH280. All strains in this study were confirmed by sequencing of confirmatory PCR products or by whole-genome sequencing using Oxford Nanopore (Plasmidsaurus).

### Growth curve generation

Growth curves were generated as previously described (9). Briefly, strains were precultured in ATCC 550 + 20 mM malate medium and LB medium combined at a 1:1 ratio at 30°C and 225 RPM shaking for 18–24 h. After preculture growth, OD_600_ was measured, and cultures were normalized to an OD_600_ = 1 by washing and resuspending cells in ATCC 550 medium without carbon. Two microliters of normalized precultures was used to inoculate 198 µL of ATCC 550 medium + indicated carbon substrate in wells of a 100-well microtiter plate. Unless otherwise noted, cells were grown on 40 mM acetate, 20 mM propionate, 20 mM butyrate, 20 mM valerate, or 20 mM hexanoate. Carbon concentrations were chosen to provide similar lag times and growth rates across substrates. Cells were grown at 30°C with continuous orbital shaking, and OD_600_ measurements were taken every 15 min in a Bioscreen C Pro microplate reader (Growth Curves USA). Maximum growth rate (*µ*_max_) was calculated using the R package QurvE using default settings for non-parametric fit (45).

### qRT-PCR

*C. necator* strains ERH090 and ERH238 were inoculated into ATCC 550 + 20 mM malate medium and LB medium combined at a 1:1 ratio and grown at 30°C with 225 RPM shaking. After 18 h of growth, cultures were centrifuged at 4,000 *× g* for 10 min, washed, resuspended in an equal volume of ATCC 550 + 20 mM hexanoate, and incubated at 30°C for 5 h. After incubation, 0.5 mL of each culture was harvested, and total RNA was extracted using a Zymo Quick-RNA Miniprep Kit according to the manufacturer’s instructions. Purified RNA was subsequently used for qRT-PCR using Luna Universal One-Step RT-qPCR Master Mix (New England Biolabs) according to the manufacturer’s instructions. This included 10 min at 55°C for reverse transcription, 95°C for 1 min for initial denaturation, 40 cycles of 95°C denaturation for 10 s, and 60°C extension for 30 s. The primers used for quantification of *H16_B1334*, *H16_B1335*, and *H16_A0005* can be found in Table S6. The relative gene expression was calculated using the delta-delta Ct method using *H16_A0005* as a reference gene (46). *H16_A0005* was chosen as a reference gene due to its consistent expression across strains and conditions in this study (Tables S1 and S2) and in our previously published work (9).

### ACS enzyme expression and purification

The most highly overexpressed ACS genes in our RNA sequencing data set were selected for heterologous expression and purification (Fig. S4C). Plasmids containing ACS genes translationally fused to an N-terminal hexahistidine tag were constructed by Twist Biosciences and transformed into an *E. coli* BL21(DE3) ∆*patZ* expression strain. The *patZ* deletion was used to prevent possible deactivating acylation of a conserved lysine residue in ACS enzymes (47). A single colony was used to inoculate 25 mL LB medium supplemented with 50 µg/mL kanamycin and grown to stationary phase overnight at 37°C. Unless otherwise indicated, expression cultures were grown to mid-log phase in 1 L terrific broth medium containing 50 µg/mL kanamycin at 37°C. When cultures reached mid-log phase, 1 mM isopropyl-β-D-thiogalactopyranoside (IPTG) was added, and enzymes were expressed at 30°C for 48 h. To improve expression, *H16_B1102* was expressed with 0.1 mM IPTG at 18°C for 20 h. In addition to enzymes with data reported, we also attempted to express *H16_A0285* and *H16_A1358*, but these enzymes did not express well in our hands. Cells were harvested by centrifugation, and pellets were stored at −80°C until purification.

Unless otherwise indicated, cell pellets were thawed to room temperature and resuspended in lysis buffer (50 mM Tris, 150 mM NaCl, 10 mM imidazole, pH 7.5). Next, 1.0 mg/mL lysozyme and 10 µg/mL DNase were added, and cells were lysed by sonication. The resulting lysate was subsequently clarified by centrifugation. For purification, the soluble protein fraction was incubated with pre-equilibrated Ni-nitrilotriacetic acid (NTA) agarose resin, washed with wash buffer (25 mM imidazole in PBS, pH 8.0), and eluted with elution buffer (50 mM imidazole in PBS, pH 8.0). Elution fractions were combined and concentrated using 10 kDa molecular weight cutoff centrifugal filters (Amicon Ultra) and further purified by size-exclusion chromatography (SEC) with 50 mM Tris buffer at pH 8.0. Purified proteins were flash-frozen in liquid nitrogen and stored at −80°C until *in vitro* activity assays. Protein concentration was assessed by absorbance at 280 nm, and purity was assessed by SDS-PAGE and ImageJ analysis (Fig. S4A; Table S9).

Purification of stable and soluble *H16_B1335* enzyme required the presence of 10% glycerol. Buffers for *H16_B1335* lysis and Ni-NTA purification were modified to include the following: 50 mM Tris, 200 mM NaCl, and 10% glycerol at pH 7.5. 10, 25, and 250 mM imidazole added to lysis, wash, and elution buffers, respectively. SEC was not performed for *H16_B1335*; Ni-NTA elution fractions were instead dialyzed against 50 mM Tris, 200 mM NaCl, 10% glycerol, and pH 7.5 to remove imidazole.

### In vitro ACS enzyme activity assays

An enzyme-coupled assay was used to measure ATP hydrolysis upon substrate thioesterification with CoA by monitoring the conversion of NADH to NAD^+^ spectroscopically (48). Assay components were as follows: 0.5–10 µg ACS enzyme; 2 mM VFA substrate; 1 mM CoA; 2.1 mM ATP; 5 mM MgCl_2_; 5 mM tris(2-carboxyethyl)phosphine; 10 U each of myokinase, pyruvate kinase, and lactate dehydrogenase; 5 mM phosphoenolpyruvate; 2 mM NADH; 100 mM Tris buffer, pH 7.5; and 100 µL total volume. The enzyme was pre-incubated with the VFA substrate, and the reaction was initiated by adding a master mix containing the remaining reagents. The decrease in NADH absorbance was monitored at 340 nm for 1 h using a Synergy H1 plate reader. The NADH concentration was calculated from a standard curve, and the extent of ATP hydrolysis was determined from the fact that two moles of NADH are consumed for every mole ATP hydrolyzed. The slope of the linear range was used to determine specific enzyme activity (µM ATP consumed/µg protein/min), and all data points were background-corrected using a no-substrate negative control. Protein yield and purity varied for each enzyme, so we chose to normalize specific activity values (Fig. S4E) to the average specific activity (µM ATP consumed/µg protein/min) on the most active substrate for each enzyme (Fig. 3).

## Data Availability

Unprocessed RNA sequencing data can be found in the National Center for Biotechnology Information Sequence Read Archive (NCBI SRA) under BioProject ID PRJNA1216626. Plasmids pSLB024, pSLB025, pSLB026, pSLB027, pSL028, pSLB029, and pSLB030 have been deposited to Addgene (IDs 234633-234639). Strains generated during this study are available upon request.

## SUPPLEMENTAL MATERIAL

Supplemental figures aem.00515-25-s0001.docx Figures S1 to S10.

Supplemental tables aem.00515-25-s0002.xlsx Tables S1 to S9.

ASM does not own the copyrights to Supplemental Material that may be linked to, or accessed through, an article. The authors have granted ASM a non-exclusive, world-wide license to publish the Supplemental Material files. Please contact the corresponding author directly for reuse.
