# Supplementary material for: Insights into genetic determinants of volatile fatty acid catabolism in Cupriavidus necator H16
Source: Appl Environ Microbiol. 2025 Jun 12;91(7):e00515-25. doi: 10.1128/aem.00515-25 (PMC12285253; doi:10.1128/aem.00515-25)
Supplement: Supplemental figures — Figures S1 to S10. [file aem.00515-25-s0001.docx]

**SUPPLEMENTAL FIGURES**


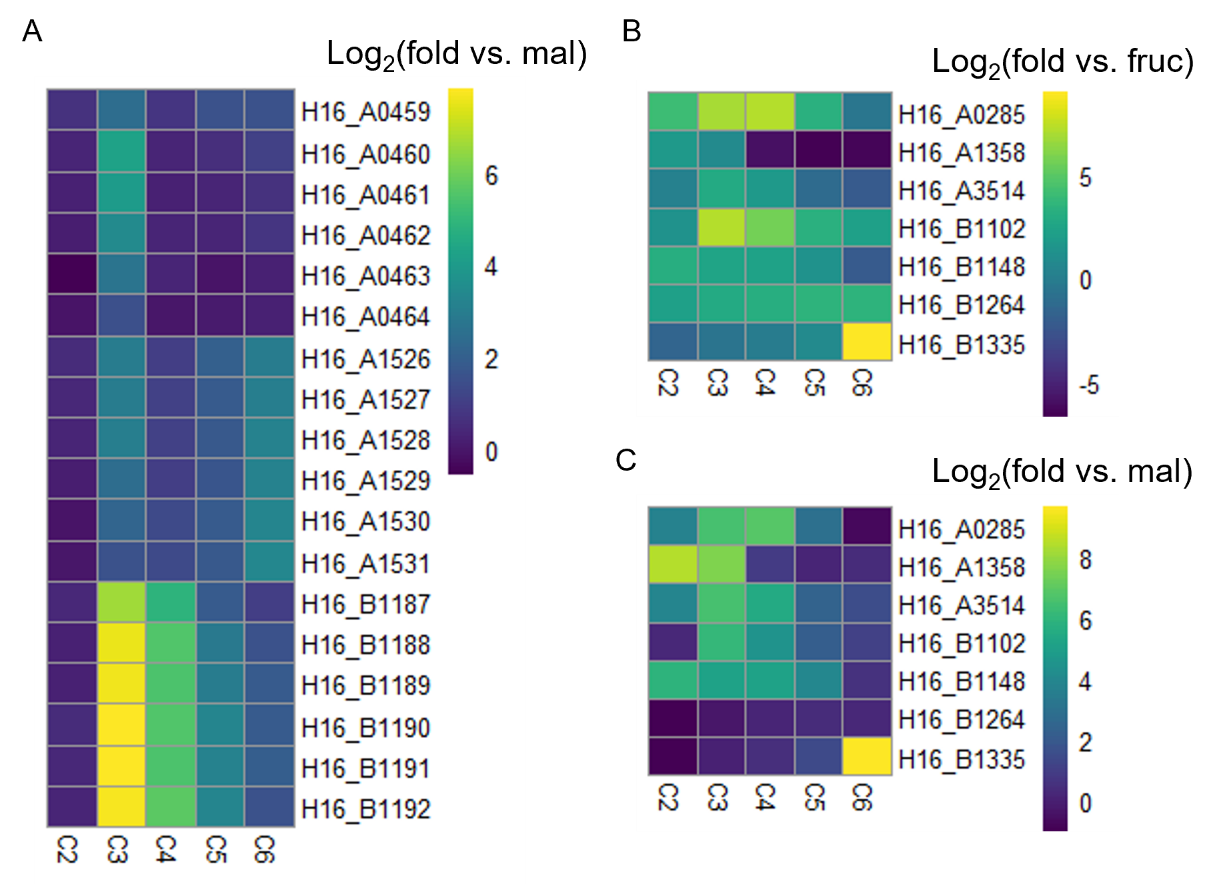


**Figure S1 | (A)** Expression heatmap of operons previously shown to be involved in fatty acid catabolism (1-3). Each heatmap column represents log-transformed gene expression for the indicated substrate (C2 = acetate, C3 = propionate, C4 = butyrate, C5 = valerate, C6 = hexanoate) compared to malate as a control. All log-transformed relative expression values are plotted on a linear gradient from -0.6 to 7.8 as indicated in the legend on the right of the figure. **(B)** Expression heatmap for selected ACS- and ACT-encoding genes with high overexpression on one or more VFA substrates. Each heatmap column represents log-transformed gene expression for the indicated substrate (C2 = acetate, C3 = propionate, C4 = butyrate, C5 = valerate, C6 = hexanoate) compared to fructose as a control. All log-transformed relative expression values are plotted on a linear gradient from -6.8 to 9 as indicated in the legend on the right of the figure. **(C)** Expression heatmap for selected ACS- and ACT-encoding genes with high overexpression on one or more VFA substrates. Each heatmap column represents log-transformed gene expression for the indicated substrate (C2 = acetate, C3 = propionate, C4 = butyrate, C5 = valerate, C6 = hexanoate) compared to malate as a control. All log-transformed relative expression values are plotted on a linear gradient from -1 to 8.5 as indicated in the legend on the right of the figure.


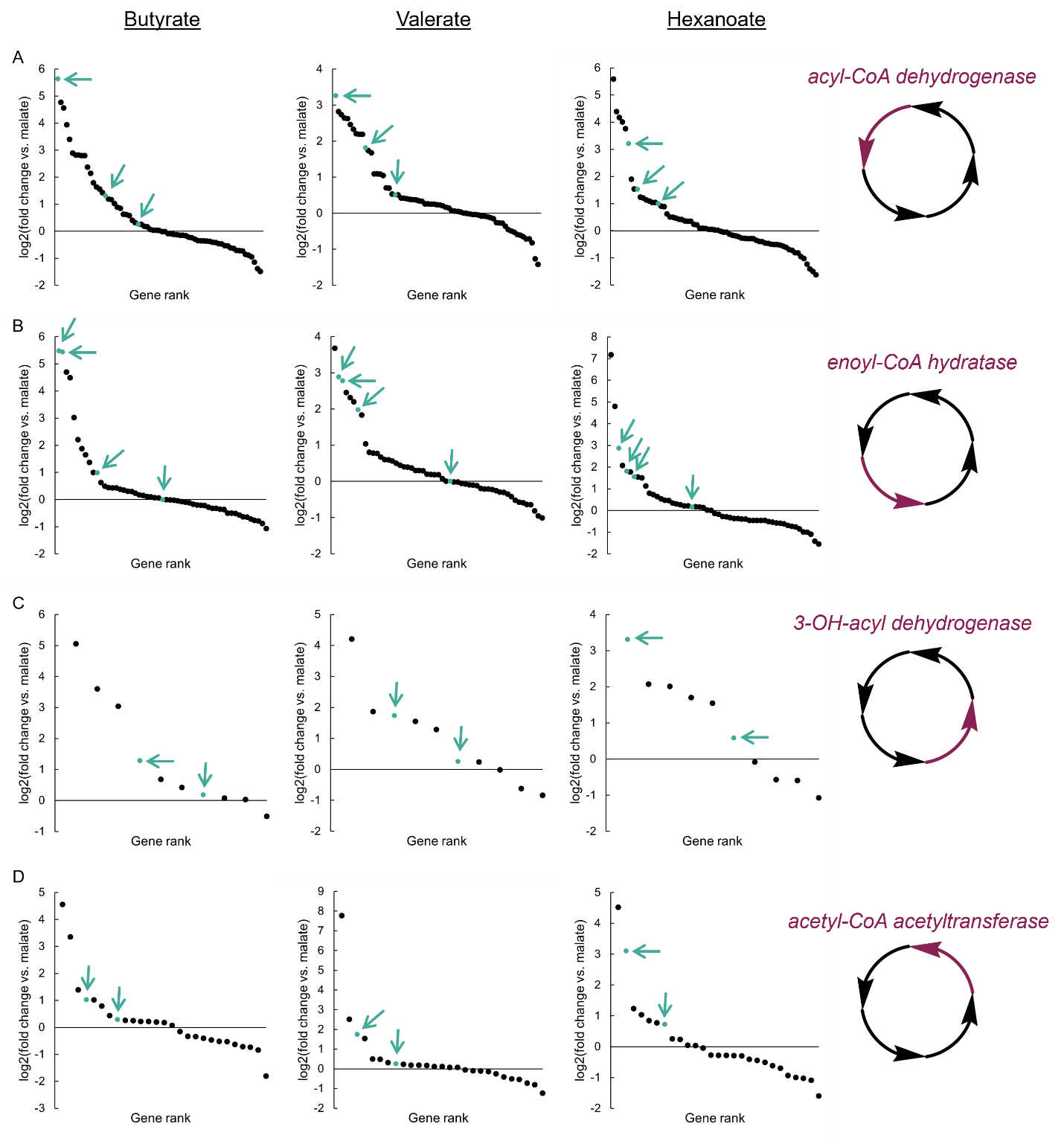


**Figure S2 |** Log-transformed fold change expression differences during growth on butyrate (left graphs), valerate (center graphs), and hexanoate (right graphs) compared to malate as a control for homologs of the *β*-oxidation genes **(A)** acyl-CoA dehydrogenase, **(B)** enoyl-CoA hydratase, **(C)** 3-OH-acyl dehydrogenase, and **(D)** acetyl-CoA acetyltransferase. Each dot represents an individual gene homolog and genes are rank-ordered by log-transformed expression. Dots colored in teal and marked with teal arrows represent genes known to be involved in *β*-oxidation (H16_A0460-A0464, H16_A1526-A1531, H16_B1188-B1192) (1-3).


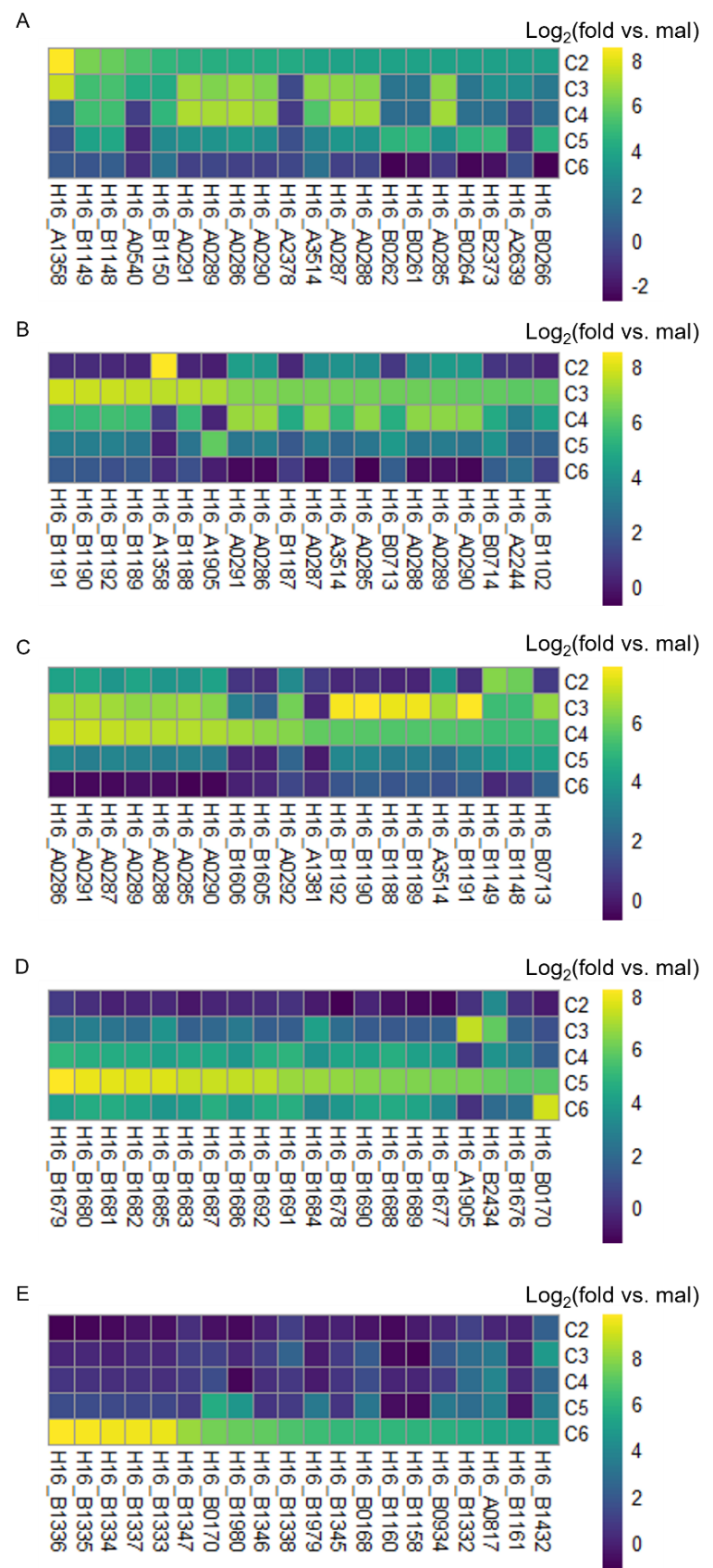


**Figure S3 |** Expression heatmaps of the most highly overexpressed genes in CHC123 when grown on VFA substrates. Each heatmap panel contains data for the top 20 most overexpressed genes when *C. necator* was grown on an individual substrate: **(A)** top 20 overexpressed genes on acetate (C2), **(B)** propionate (C3), **(C)** butyrate (C4), **(D)** valerate (C5), and **(E)** hexanoate (C6) compared to malate. Genes in each heatmap are ordered left to right by overexpression on the respective substrate and each row represents log-transformed gene expression for the indicated substrate (C2 = acetate, C3 = propionate, C4 = butyrate, C5 = valerate, C6 = hexanoate) compared to malate as a control**.**


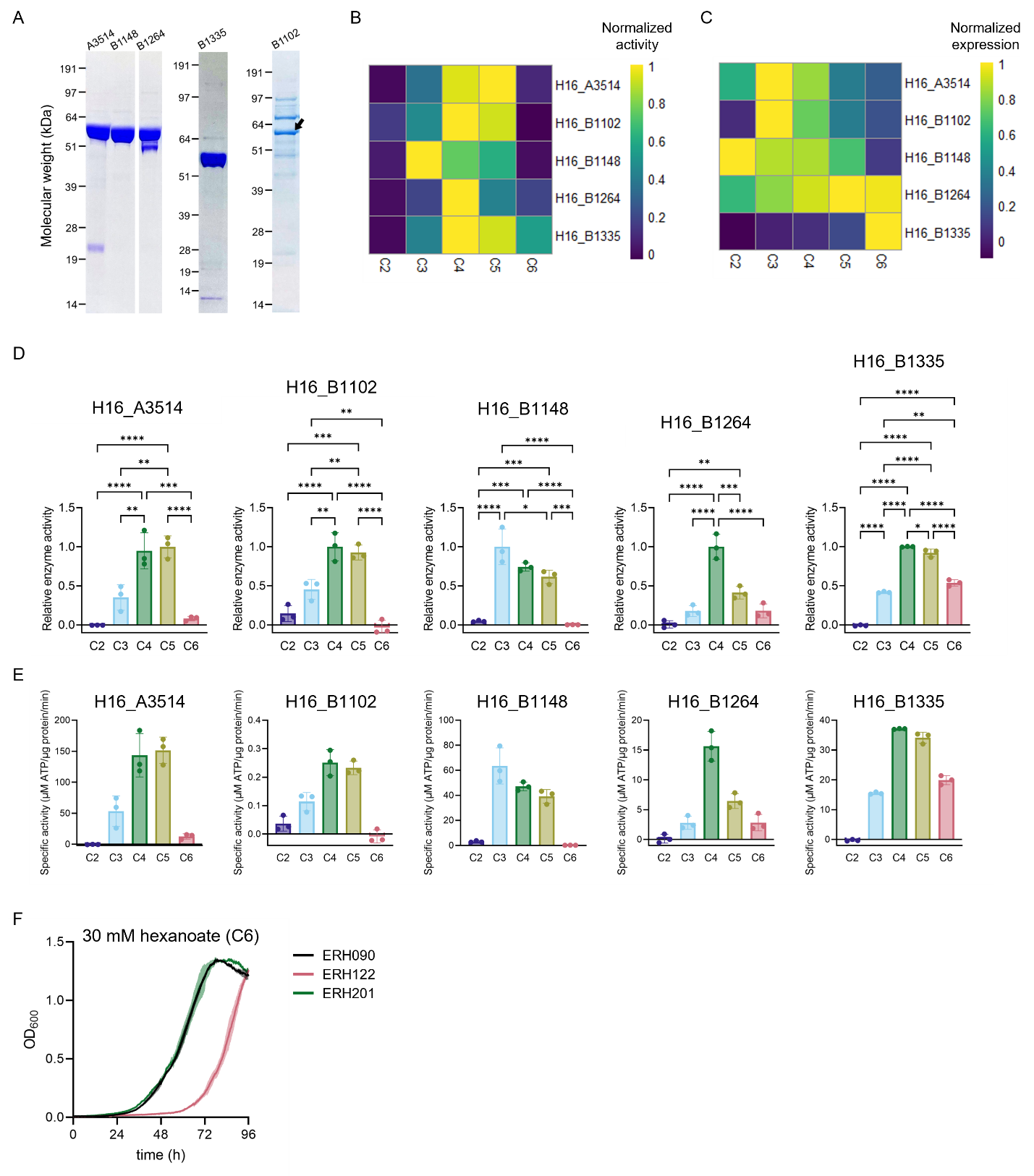


**Figure S4** | **(A)** SDS-PAGE of purified ACS products produced in *E. coli.* Arrow indicates expected size of H16_B1102. Percent purity of enzymes was calculated using ImageJ and values are summarized in **Table S9.** **(B)** Heatmap of normalized *in vitro* enzyme activity on acetate (C2), propionate (C3), butyrate (C4), valerate (C5), or hexanoate (C6). Activity is normalized to the top specific activity for each individual ACS enzyme. **(C)** Heatmap of normalized overexpression of ACS enzymes in CHC123 when grown on acetate (C2), propionate (C3), butyrate (C4), valerate (C5), or hexanoate (C6). Values are normalized to the top log_2_(fold change expression) compared to malate (*H16_A3514*, *H16_B1102*, *H16_B1148*, *H16_B1335*) or fructose (*H16_B1264*). **(D)** Relative activity of ACS enzymes purified from *E. coli* and tested *in vitro* on the substrates acetate (C2, dark blue bars), propionate (C3, light blue bars), butyrate (C4, green bars), valerate (C5, gold bars), and hexanoate (C6, pink bars). Values are normalized by the average specific activity (µM ATP consumed/µg protein/min) of the most active substrate for each respective enzyme. Bars represent the average and error bars the standard deviation of biological triplicates for each ACS enzyme, as indicated above the panel. Data plotted is the same as in **Figure 3**, with statistical comparisons overlaid. Asterisks denote statistical significance (one-way ANOVA, Tukey’s test, ** P* < 0.05, ** *P* < 0.01, *** *P* < 0.001, **** *P* < 0.0001). **(E)** Specific activity (µM ATP consumed/µg protein/min) of ACS enzymes produced in *E. coli* and tested *in vitro* on the substrates acetate (C2, dark blue bars), propionate (C3, light blue bars), butyrate (C4, green bars), valerate (C5, gold bars), and hexanoate (C6, pink bars). Bars represent the average and error bars the standard deviation of biological triplicates for each ACS enzyme, as indicated above the panel. **(F)** Growth, measured as OD_600_, of ERH090 (black curve), ERH122 (pink curve), and ERH201 (green curve) grown on 30 mM hexanoate. Lines represent the average and shading the standard deviation of biological triplicates.


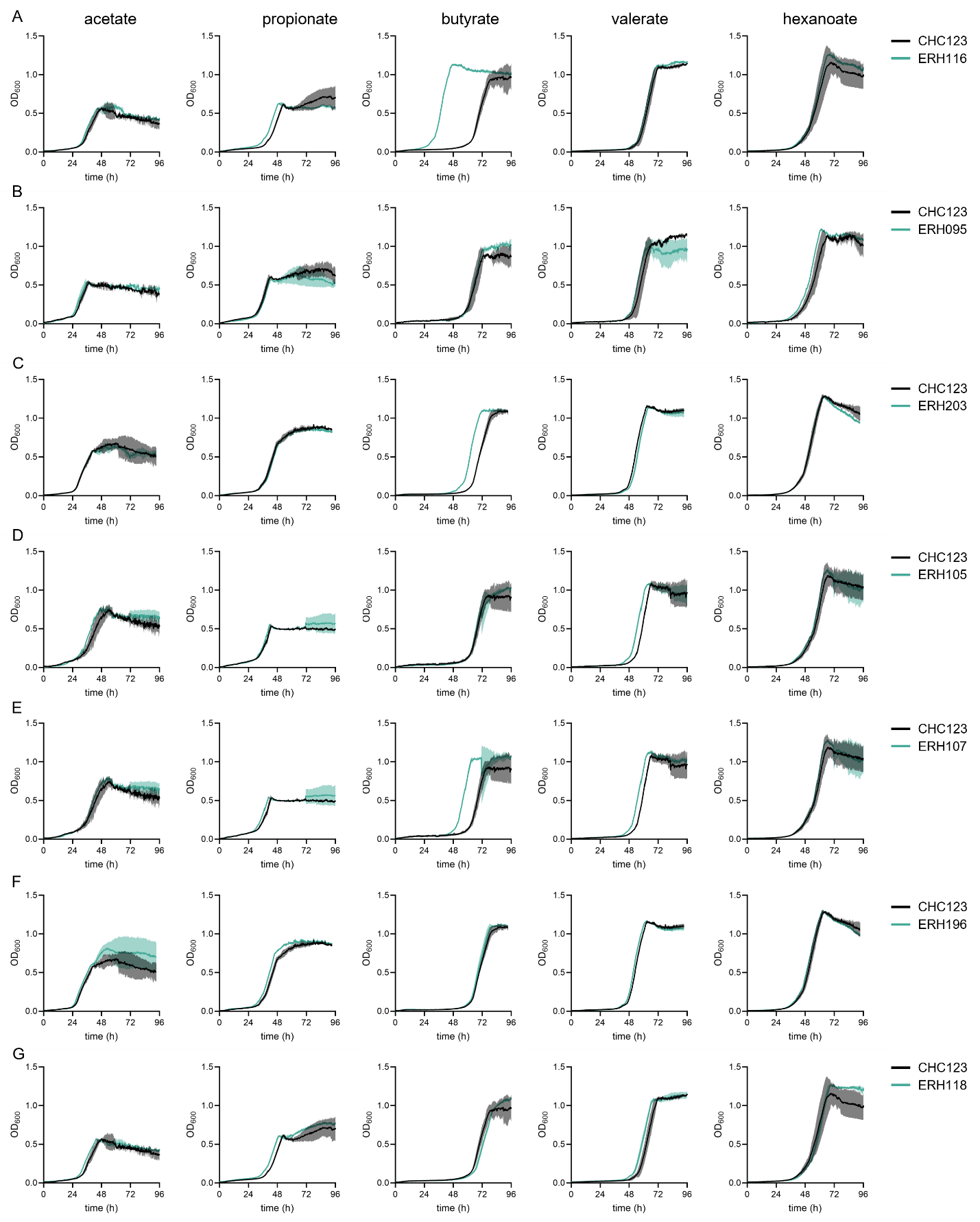


**Figure S5 |** Growth, measured as OD_600_, of CHC123 (black curves) and in teal curves **(A)** ERH116, **(B)** ERH095, **(C)** ERH203, **(D)** ERH105, **(E)** ERH107, **(F)** ERH196, and **(G)** ERH118 grown on acetate, propionate, butyrate, valerate, and hexanoate as indicated above individual graphs columns. Lines represent the average and shading the standard deviation of biological triplicates.


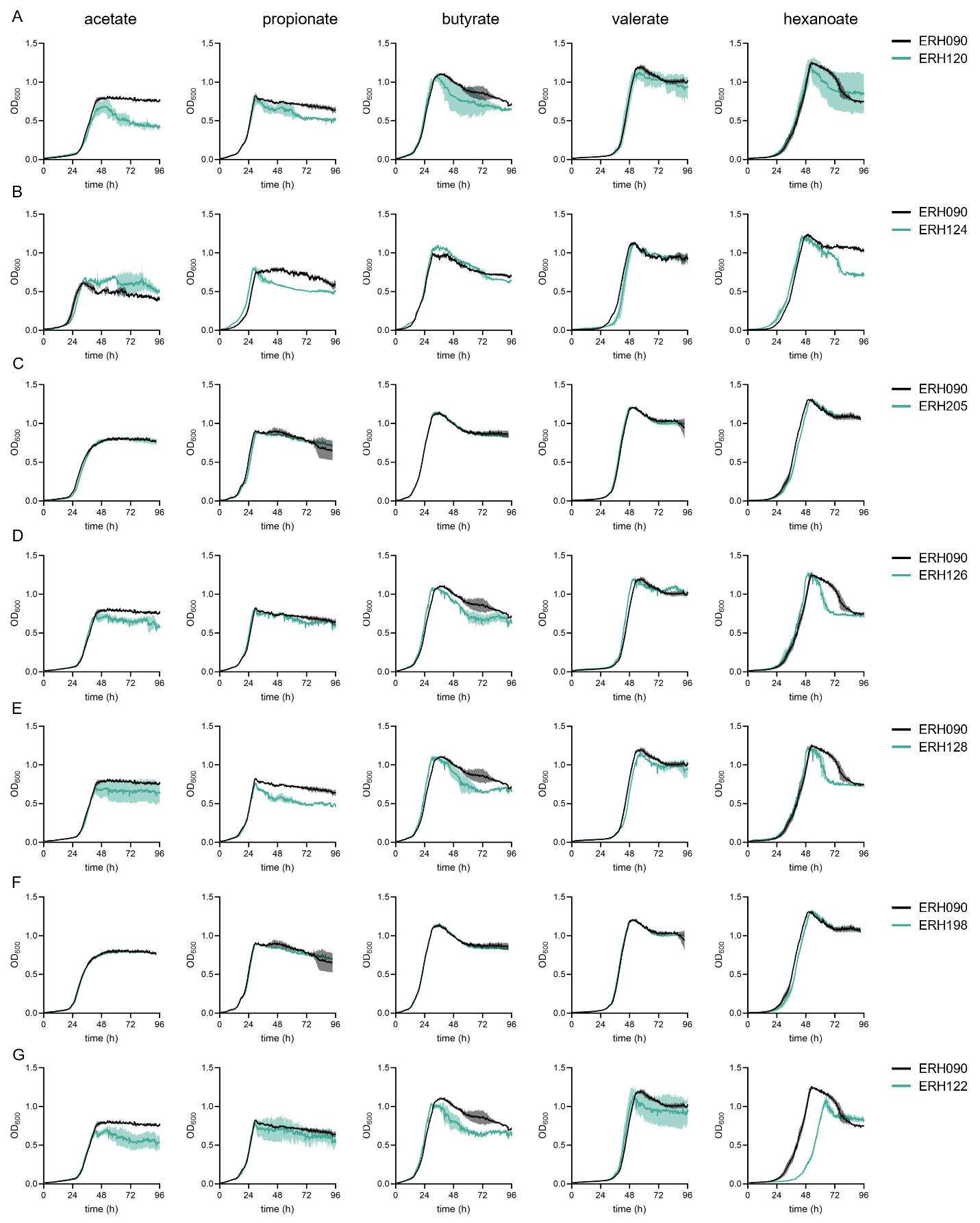


**Figure S6 |** Growth, measured as OD_600_, of ERH090 (black curves) and in teal curves **(A)** ERH120, **(B)** ERH124, **(C)** ERH205, **(D)** ERH126, **(E)** ERH128, **(F)** ERH198, and **(G)** ERH122 grown on acetate, propionate, butyrate, valerate, and hexanoate as indicated above individual graphs columns. Lines represent the average and shading the standard deviation of biological triplicates.


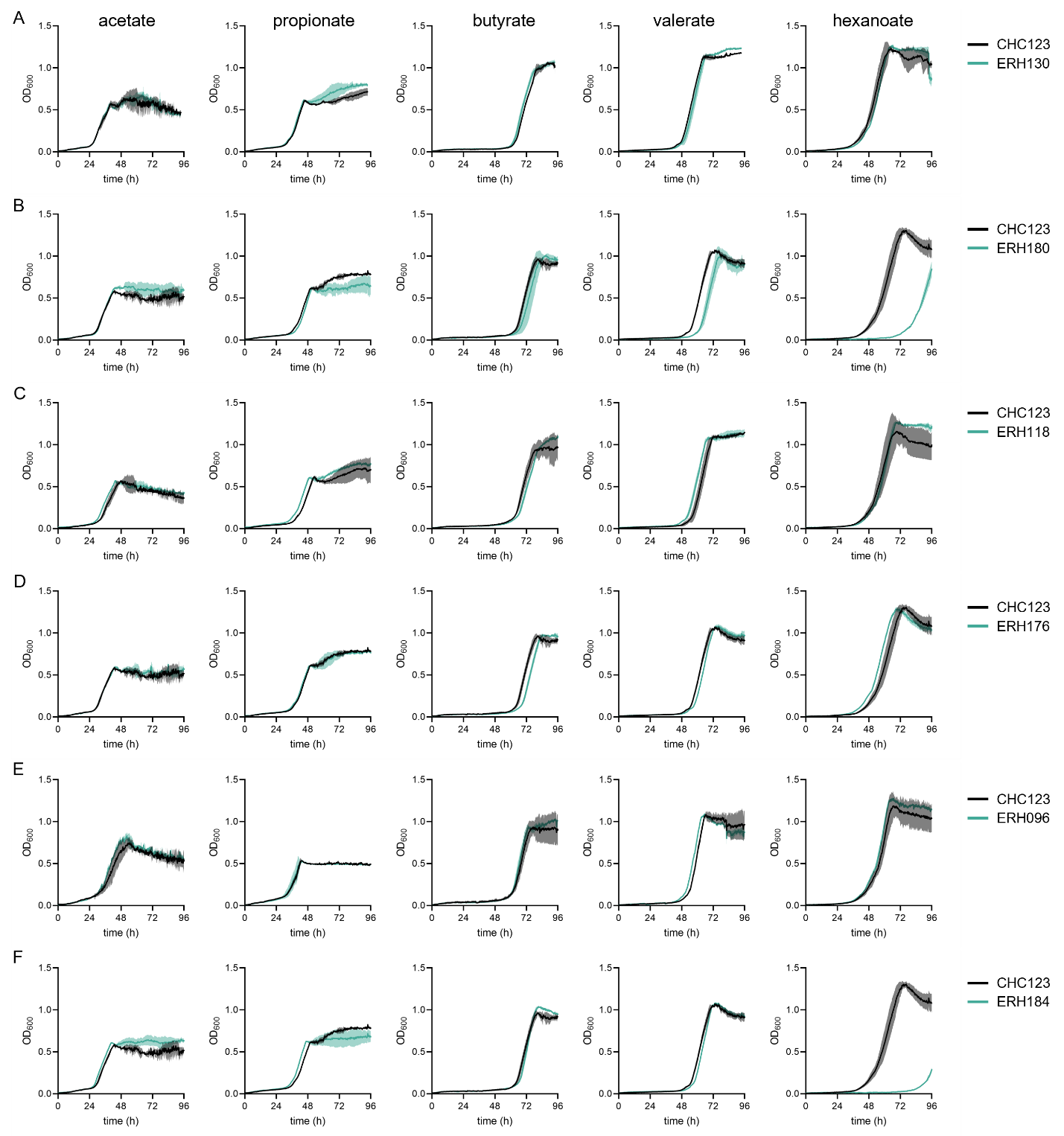


**Figure S7** | Growth, measured as OD_600_, of CHC123 (black curves) and in teal curves **(A)** ERH130, **(B)** ERH180, **(C)** ERH118, **(D)** ERH176, **(E)** ERH096, and **(F)** ERH184 grown on acetate, propionate, butyrate, valerate, and hexanoate as indicated above individual graphs columns. Lines represent the average and shading the standard deviation of biological triplicates.


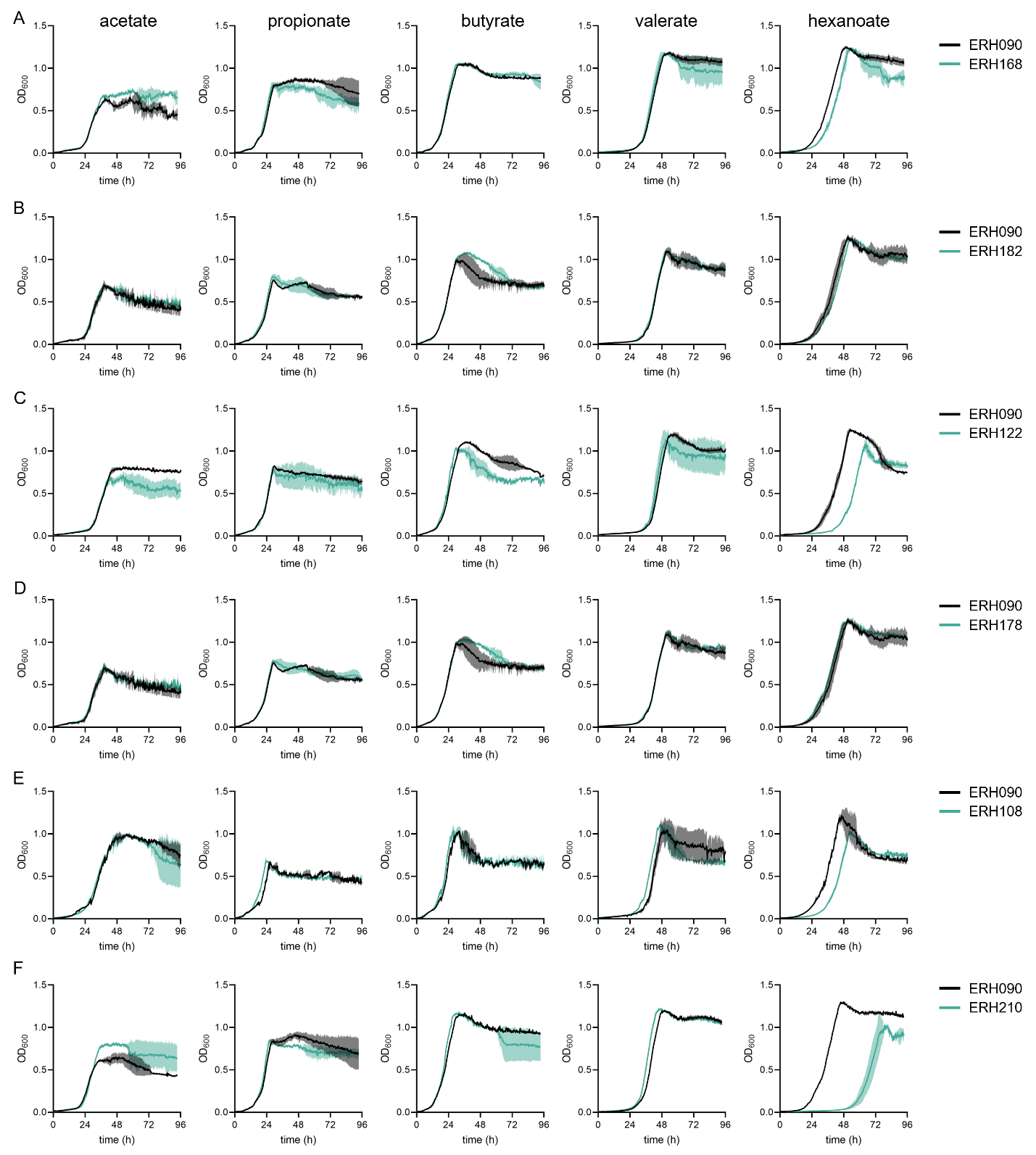


**Figure S8 |** Growth, measured as OD_600_, of ERH090 (black curves) and in teal curves **(A)** ERH168, **(B)** ERH188, **(C)** ERH122, **(D)** ERH178, **(E)** ERH108, and **(F)** ERH210 grown on acetate, propionate, butyrate, valerate, and hexanoate as indicated above individual graphs columns. Lines represent the average and shading the standard deviation of biological triplicates.


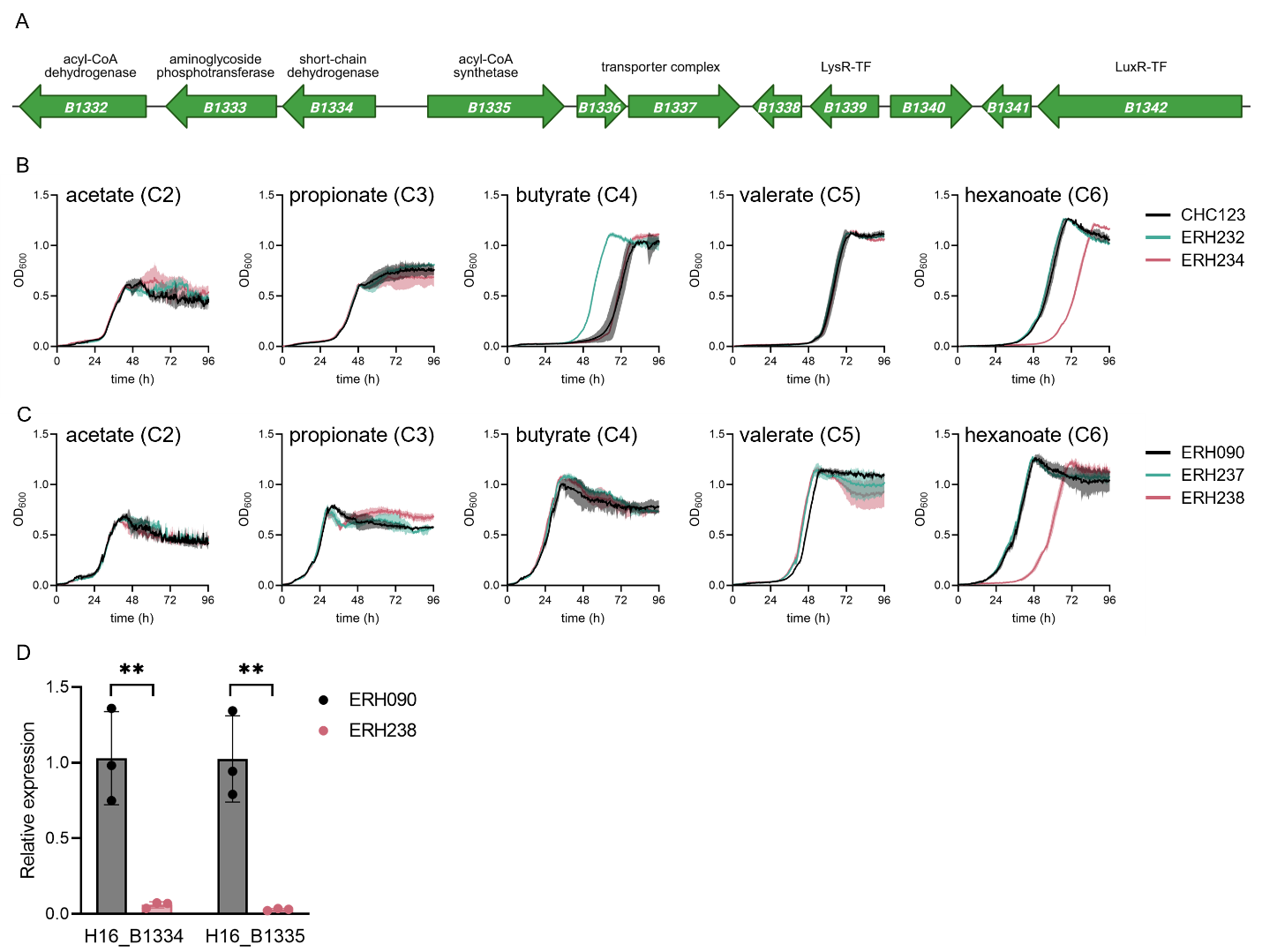


**Figure S9 | (A)** Structure of the *H16_B1332-H16_B1342* genetic loci, including putative gene annotations. **(B)** Growth, measured as OD_600_, of CHC123 (black curves), ERH232 (teal curves), and ERH234 (pink curves) grown on acetate, propionate, butyrate, valerate, and hexanoate. Lines represent the average and shading the standard deviation of biological triplicates. **(C)** Growth, measured as OD_600_, of ERH090 (black curves), ERH237 (teal curves), and ERH238 (pink curves) grown on acetate, propionate, butyrate, valerate, and hexanoate. Lines represent the average and shading the standard deviation of biological triplicates. **(D)** Relative gene expression of *H16_B1334* and *H16_B1335*, as determined by RT-qPCR, in strains ERH090 (black bars) and ERH238 (pink bars) after 5 h of incubation with hexanoate. Individual replicates are normalized to expression of the reference gene *H16_A0005* and to the average relative expression in ERH090. Bars represent the average and error bars the standard deviation of biological triplicates. Asterisks represents statistical significance (2-tailed student’s t-test, ** *P* < 0.01)


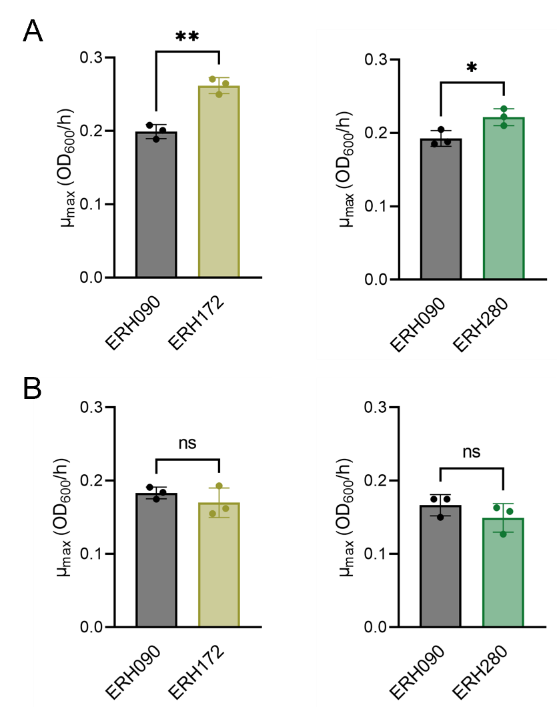


**Figure S10 | (A)** Maximum growth rate (µ_max_) of ERH090 (black bars), ERH172 (gold bar), and ERH280 (green bar) grown on valerate. **(B)** Maximum growth rate (µ_max_) of ERH090 (black bars), ERH172 (gold bar), and ERH280 (green bar) grown on hexanoate. Growth rates correspond to curves in **Figure 6**. Asterisks in (A) and (B) denote statistical significance (Student’s t-test, ns – not significant, ** P* < 0.05, ** *P* < 0.01).

**SUPPLEMENTAL REFERENCES**

1. Strittmatter CS, Eggers J, Biesgen V, Hengsbach J-N, Sakatoku A, Albrecht D, Riedel K, Steinbüchel A. 2022. Insights into the degradation of medium-chain-length dicarboxylic acids in *Cupriavidus necator* H16 reveal β-oxidation differences between dicarboxylic acids and fatty acids. Applied and Environmental Microbiology 88:e01873-21.

2. Strittmatter CS, Poehlein A, Himmelbach A, Daniel R, Steinbüchel A. 2022. Medium-chain-length fatty acid catabolism in *Cupriavidus necator* H16: transcriptome sequencing reveals differences from long-chain-length fatty acid β-oxidation and involvement of several homologous genes. Applied and Environmental Microbiology 89:e01428-22.

3. Brigham CJ, Budde CF, Holder JW, Zeng Q, Mahan AE, Rha C, Sinskey AJ. 2010. Elucidation of β-oxidation pathways in *Ralstonia eutropha* H16 by examination of global gene expression. Journal of Bacteriology 192:5454-5464.
